# Ultrafast strain propagation and acoustic resonances in nanoscale bilayer systems

**DOI:** 10.1063/4.0000079

**Published:** 2021-06-18

**Authors:** N. Bach, S. Schäfer

**Affiliations:** Institute of Physics, University of Oldenburg, 26129 Oldenburg, Germany

## Abstract

Ultrafast structural probing has greatly enhanced our understanding of the coupling of atomic motion to electronic and phononic degrees-of-freedom in quasi-bulk materials. In bi- and multilayer model systems, additionally, spatially inhomogeneous relaxation channels are accessible, often governed by pronounced interfacial couplings and local excitations in confined geometries. Here, we systematically explore the key dependencies of the low-frequency acoustic phonon spectrum in an elastically mismatched metal/semiconductor bilayer system optically excited by femtosecond laser pulses. We track the spatiotemporal strain wave propagation in the heterostructure employing a discrete numerical linear chain simulation and access acoustic wave reflections and interfacial couplings with a phonon mode description based on a continuum mechanics model. Due to the interplay of elastic properties and mass densities of the two materials, acoustic resonance frequencies of the heterostructure significantly differ from breathing modes in monolayer films. For large acoustic mismatch, the spatial localization of phonon eigenmodes is derived from analytical approximations and can be interpreted as harmonic oscillations in decoupled mechanical resonators.

Advanced technological applications ranging from heat management in nanoelectronics^1^ to optomechanical resonators^2^ are based on nanoscale systems with engineered thermal and acoustic interfaces.^3–6^ Nanostructures like gratings,^7–9^ semiconductor quantum wells,^10^ and superlattices^11–13^ combined with sophisticated optical control strategies allow for coherent phononic and photonic excitations, and tailored infrared optical near-fields.^14,15^

The complex microscopic mechanisms of pico- and femtosecond phononic processes are being uncovered by high-resolution optical spectroscopy and recently developed ultrafast methodologies. For example, phonon–phonon couplings, modified phonon dispersion relations, and dissipation times in nanoscale systems^16–19^ are accessible by all-optical technologies such as Brillouin scattering^18,20,21^ and ultrafast pump–probe^22^ and multidimensional^23,24^ spectroscopies. Experimental approaches explicitly capturing structural deformations within individual materials with high spatial resolution in reciprocal space include ultrafast electron^25–36^ and x-ray diffraction^37–41^ techniques, extendable to time-resolved local diffractive probing using convergent electron beams.^42–44^ Furthermore, real-space imaging of photoexcited localized vibrations, propagating strain waves, and structural phase transitions is provided by time-resolved scanning probe techniques^45–47^ and ultrafast transmission electron microscopy (UTEM).^48–51^ As prototypical nanophononic sample systems, ultrafast dynamics in bilayer films^52–54^ and thin-films on semi-infinite substrates^22,55–60^ were studied in detail, focusing on the influence of interfaces^52,61,62^ and revealing unexpected interlayer electron–phonon interactions.^63–65^ Ultrafast strain dynamics in these systems are typically modeled by numerical approaches including one-dimensional linear chain^66–69^ and two-dimensional finite-element simulations.^44,70,71^ Although complex strain dynamics are accurately recaptured in such approaches, analytical models offer an additional highly valuable and intuitive physical understanding,^72–74^ but were not systematically employed for analyzing the ultrafast structural response in nanoscale heterostructures.

Here, we apply an analytical acoustic mode description of ultrafast structural dynamics in a nanoscale bilayer system. Explicit expressions for the resonance frequencies are derived and compared to numerical results. The coupling of individual phononic modes in both layers is analyzed with respect to the acoustic mismatch and the emergence of localized modes is demonstrated.

In order to arrive at a resonant acoustic mode description for bilayer films, we start off by considering a simple monolayer, as often investigated in ultrafast diffraction experiments.^26,30,41,44,75–79^ In response to a homogeneous ultrashort laser excitation of such a sample, a transient stress gradient is induced in the depth of the film causing longitudinal strain waves to travel back and forth between the free surfaces. The resulting homogeneous compression and expansion of the film is often termed breathing mode. As an example, we depict in Fig. 1(b) (top) the optically induced dynamics of a platinum thin film after excitation (see the supplementary material S1 for details). The resulting oscillatory change in film thickness occurs with a periodicity given by the strain pulse round trip time.

**FIG. 1.**
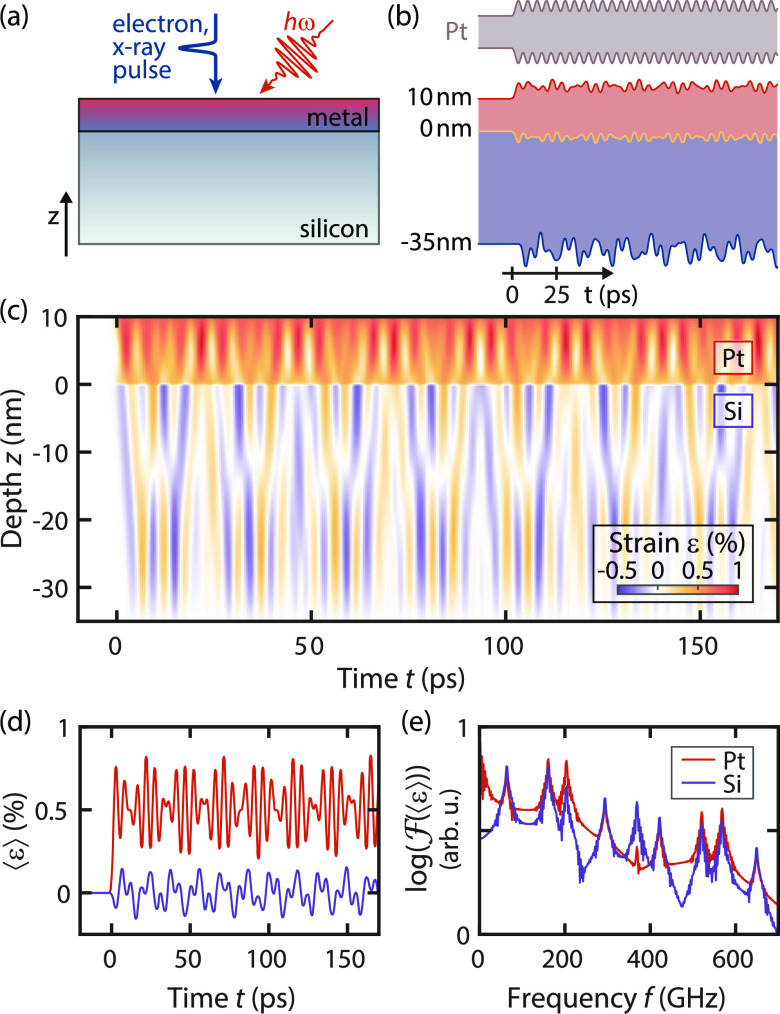
Strain dynamics in a nanoscale Pt/Si-bilayer film numerically simulated by a discrete one-dimensional linear chain model. (a) A bilayer membrane is optically excited by femtosecond laser pulses inducing a rapid lattice temperature increase and launching strain waves in both materials while silicon remains at room temperature. Structural dynamics are typically probed by ultrafast diffraction experiments harnessing the high spatial resolution in reciprocal space. (b) Temporal evolution of the surface and interface positions (displacements are amplified by a factor of 110 for better visibility) in single-layer platinum and the Pt/Si-bilayer. (c) Spatiotemporal strain map of structural dynamics induced by optical excitation at *t* = 0. (d) Mean strain 〈ε〉 in Pt (red) and Si (violet) obtained by spatially averaging the strain maps in panel (c). (e) Acoustic resonance frequencies obtained from Fourier transform of the temporal evolution of the mean strain shown in panel (d).

In an equivalent description, the traveling wave can be decomposed into a superposition $\sum_{n}a_{n}u_{n}$ of resonant modes. For a simple thin film of thickness *H*, acoustic resonant modes with mode index n∈ℕ are given by $u_{n}(z)=\text{cos}\;(n\pi z/H)$ with mode frequencies $f_{n}=nv/(2H)$, in which *v* is the longitudinal sound velocity of the material. The base frequency *f*_1_ corresponds to the inverse round trip time of the strain wave. Mode amplitudes in the superposition depend on the effective time- and length-scale of the sample excitation. Specifically, for a homogenous excitation within the depth of the thin film and for a pump-induced stress with a rise time much larger than the considered resonance frequencies, one obtains $-2\Delta H/(\pi^{2}n^{2})$ for the *n*th mode amplitude (*n* odd; ΔH/H: average lattice strain after optical excitation), resulting in a saw-tooth-like temporal variation of the film strain.

In ultrafast diffraction experiments, the temporal evolution of the average strain along the probing direction is encoded in a periodic change of reciprocal lattice vectors leading to an angular displacement of the center-of-mass of Bragg scattering conditions. Only uneven higher harmonics of the fundamental breathing mode frequency are contributing to the average angular shift. Modes with even *n* result in zero average strain and therefore no shift in the average scattering condition. The superposition of all modes yields the strain distribution within the film and is thereby encoded in the profile of the Bragg spot (or Bragg line, for convergent beam electron diffraction). As an example, the modulation of the Bragg linewidth in laser-excited thin films was found to occur at twice the frequency of the breathing mode.^44^

Although the modes in a monolayer material are directly obtained, in a two layer system, acoustic boundary conditions at the interface play a major role and require a more detailed analysis. As an example, we numerically simulate the strain dynamics in a 10-nm thin polycrystalline platinum layer on top of a 35-nm thin single-crystalline silicon membrane ([001]-orientation along the *z*-direction) using a one-dimensional linear-chain model (detailed in supplementary material S1; for material parameters, see Table I). In our model, we consider a homogeneous excitation of the top layer by an ultrashort optical pulse [see Fig. 1(a)] and assume an optically induced thermal stress due to locally equilibrated electron and lattice systems. More general cases could be included by additional transient stress contributions,^80^ such as thermoelastic electron–phonon coupling driven by hot carriers,^56,81–83^ the deformation potential mechanism,^22,57^ coupling between strain and macroscopic electric fields in non-centro symmetric materials,^10,84^ and electrostriction in transparent solids.^85^

**TABLE I.** Material properties of the Pt/Si-bilayer employed for the numerical and analytical simulations (see Refs. 86 and 87).

	Mass *m*	Lattice constant *a*	Mass density *ρ*	Sound velocity *v*	Acoustic impedance *Z*	Layer thickness
	(u)	(Å)	(kg m^−3^)	(ms^−1^)	(MPa sm^−1^)	(nm)
Silicon	28	5.43	2329	8433	19.4	35
Platinum	195	3.92	21450	3829	82.1	10

Within the theoretical model, the obtained temporal evolution of the film thickness and interface positions after optical excitation is shown in Fig. 1(b) (bottom panel, displacements are amplified for better visibility). Although only platinum is optically excited, it is apparent that multifrequency strain dynamics are induced in both layers due to interlayer strain coupling, in contrast to the single platinum layer exhibiting only a breathing mode with a single frequency [with additional higher order harmonic contributions, Fig. 1(b) (top)]. For a more detailed analysis of the strain dynamics, we extract the spatiotemporal structure of the strain field within the film, shown as a color-coded map in Fig. 1(c). Following the optical excitation at *t* = 0, the metal layer expands and a positive strain builds up, leading, in turn, to a compression of the adjacent silicon membrane. Transmitted and reflected acoustic waves at the interfaces couple with the lattice dynamics in both layers.

Depending on the experimental geometry, ultrafast electron or x-ray diffraction experiments are sensitive to different components of the strain tensor and typically involve spatial averaging along the probing axis. In particular, for low-coherence probe beams, often only the mean strain of each layer is experimentally accessible. For the case of the bilayer sample considered here, the evolution of the mean strain in both layers is shown in Fig. 1(d). Heating of the platinum top layer leads to multifrequency oscillations around an equilibrium positive strain of about 0.5%. The non-heated silicon is compressed by the adjacent expanding platinum layer and oscillates at smaller amplitudes around zero mean strain. Further insights into the temporal strain dynamics are obtained from a Fourier transform of the mean strain yielding layer-specific multiple resonance frequencies in the GHz-regime [Fig. 1(e)], with the most prominent peaks for the present case at f=63.0, 161.1,  and 203.7 GHz. Curiously, none of these frequencies match acoustic round trip times, $2l_{\text{Si}}/v_{\text{Si}}=8.3\;\text{ps}$ ( 120.5 GHz) and $2l_{\text{Pt}}/v_{\text{Pt}}=5.2\;\text{ps}$ ( 192.3 GHz), for which $l_{\text{Si}(\text{Pt})}$ is the layer thickness and $v_{\text{Si}(\text{Pt})}$ the sound velocity for wave propagation in the *z*-direction.

To demonstrate that the origin of this apparent discrepancy is linked to the coupling of acoustic modes in both layers, we performed numerical strain dynamics simulations for metal layers with different sound velocities but a fixed mass density. The obtained frequency spectra of the mean strain, depending on the velocity ratio $r_{v}=v_{m}/v_{\text{Si}}$, are shown in Figs. 2(a) and 2(b) for the metal and silicon layer, respectively. For large velocity ratios and a fixed sound velocity of silicon, high-frequency modes are observed at frequencies related to the reciprocal round trip time in the silicon bottom layer (for details, see below). In contrast, resonance frequencies scale linearly with $v_{m}$ in the limit of small velocity ratios. In the intermediate regime, strong couplings between both layers are apparent, showing avoided crossings of resonance frequency branches and a complex pattern of changes in the resonance amplitudes.

**FIG. 2.**
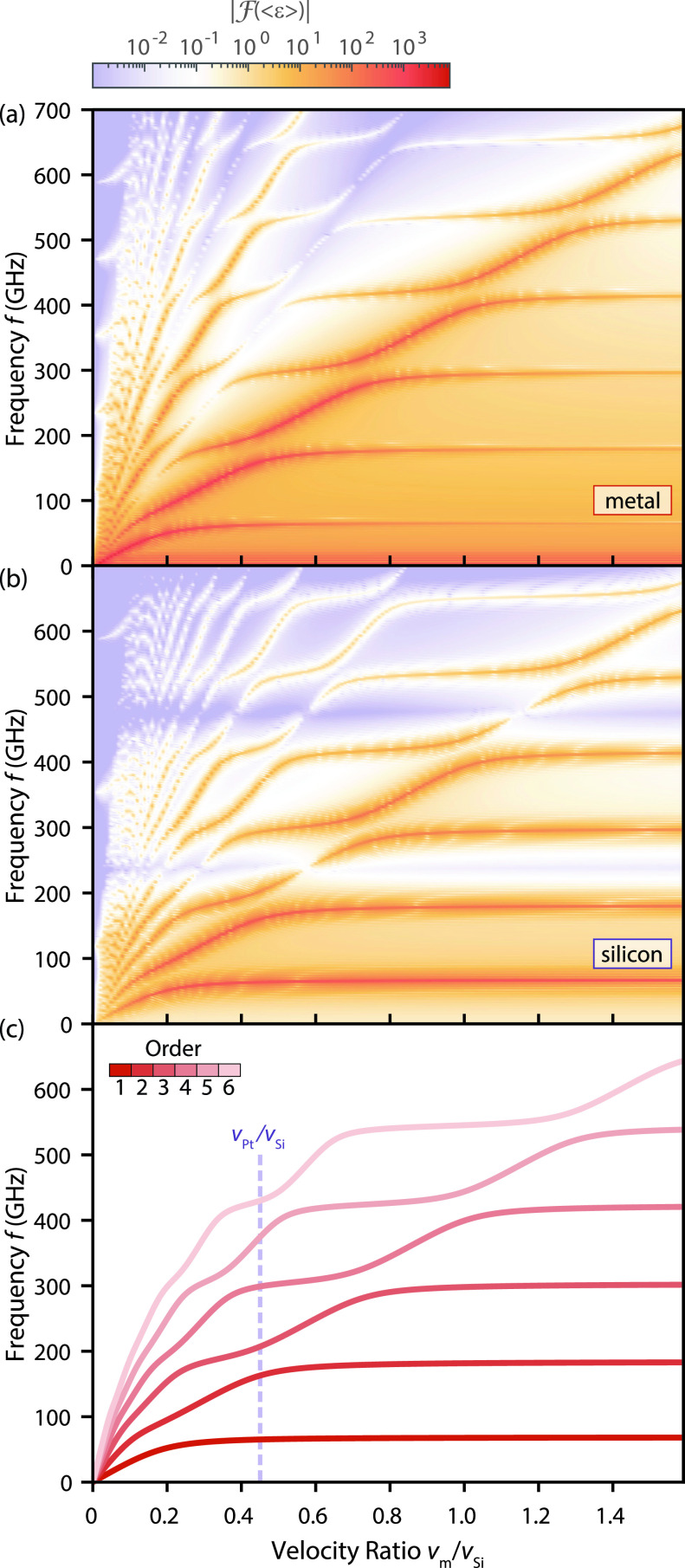
Acoustic resonance frequencies in a metal/silicon bilayer. (a) and (b) Fourier transform of the temporal evolution of the mean strain in the metal and silicon layers evaluated for different ratios of the sound velocities, $r_{v}=v_{m}/v_{\text{Si}}$. In the limits of small and large ratios, a linear scaling of the resonance frequencies or an equidistant ladder spectrum is observed, respectively. (c) Resonance frequencies derived from the roots of the characteristic function (1), in quantitative agreement with the peak locations of the Fourier spectra in panels (a) and (b).

In the following, an analytical description is presented allowing for a more general discussion of strain propagation in coupled bilayer systems. For strongly mismatched bulk material properties, approximate solutions are found yielding insights into the origin of coupling mechanisms between adjacent layers. The acoustic wave field in the two quasi-isotropic elastic media, platinum and silicon, is described as a linear combination of back and forth propagating plane waves along the *z*-direction [see Table I for material properties and Fig. 1(a) for the coordinate system of the two-dimensional cross section]. The relative wave amplitudes are determined by the acoustic boundary conditions at the interfaces between the materials and at the free surfaces, mathematically accumulated in a so-called global matrix D(ω) (see the supplementary material S2 for details). Allowed acoustic mode frequencies *ω_n_* are obtained as the *n*th order root of the resulting characteristic function,

$$\begin{array}{c} \det\left[D(\omega )\right]=\text{sin}\;\left(\omega \frac{H_{\text{Si}}}{v_{\text{Si}}}\right)\text{cos}\;\left(\omega \frac{H_{m}}{v_{m}}\right)\\ -\frac{Z_{m}}{Z_{\text{Si}}}\text{cos}\;\left(\omega \frac{H_{\text{Si}}}{v_{\text{Si}}}\right)\text{sin}\;\left(\omega \frac{H_{m}}{v_{m}}\right)\overset{!}{=}0, \end{array}$$ (1)

in which $H_{\text{Si}}$ and $H_{m}$ are the positions of the silicon/vacuum and metal/vacuum interfaces. The impedances $Z_{\text{Si},m}$ are given by $\rho_{\text{Si},m}v_{\text{Si},m}$. The lowest-order roots $f^{\left(n\right)}=\omega^{\left(n\right)}/(2\pi )$ for varying velocity ratios *r_v_* are plotted in Fig. 2(c) for n∈1,…,6, reproducing the resonance branch structure retrieved from the linear-chain model.

The resonance frequencies in the limit of large ratios ( $v_{m}/v_{\text{Si}}\to \infty$) are understood by a first-order Taylor expansion of the characteristic function, yielding $\text{tan}\;\left(\omega \frac{H_{\text{Si}}}{v_{\text{Si}}}\right)=\frac{\rho_{m}}{\rho_{\text{Si}}}\frac{H_{m}}{v_{\text{Si}}}\omega$. In this limit and for large frequencies *ω*, solutions asymptotically approach the poles of the tangent functions located at $f^{\left(n\right)}=(n-\frac{1}{2})\frac{1}{T_{\text{Si}}}$ with $T_{\text{Si}}=\frac{2H_{\text{Si}}}{v_{\text{Si}}}$. The frequency branches of low mode order, particularly the first root of the characteristic function, significantly deviate from the frequency expected from the round trip time in silicon. Similarly for small velocity ratios ( $v_{m}/v_{\text{Si}}\to 0$), $f^{\left(n\right)}=(n-\frac{1}{2})\frac{v_{m}}{2H_{m}}$ is obtained.

A change in *r_v_* results in both a varying round trip time and a change in the mode coupling. To disentangle both contributions, we consider in the following a density variation in the metal layer, keeping its sound velocity constant. Thereby, the round trip time in the metal layer is constant but the relative acoustic impedance *r_Z_* of both layers changes. The resulting roots $f^{\left(n\right)}(r_{Z})$ of the characteristic function are plotted in Fig. 3(a). For impedance matched layers, i.e., $\text{log}\;(Z_{m}/Z_{\text{Si}})=0$, the acoustic wave propagates without reflection at the interface yielding equal wave amplitudes in both layers. In this case, the characteristic function simplifies to $\text{sin}\;\left[\left(\frac{\left|H_{\text{Si}}\right|}{v_{\text{Si}}}+\frac{H_{m}}{v_{m}}\right)\omega \right]=0$, resulting in resonance frequencies $f^{\left(n\right)}=\frac{n}{T_{m}+T_{\text{Si}}}$ with n∈ℕ. In the limiting cases of small and large impedance ratios, the roots of the characteristic function can be also analytically obtained (see the supplementary material S2 for corresponding Taylor expansions). Specifically, for $r_{Z}\to 0$, the resonance frequencies are given by $nT_{\text{Si}}$ and $(n-\frac{1}{2})T_{m}$, so that the frequency spectrum is formed by two equidistant ladder spectra, indicated as (n)-Si and (n)-Pt in Fig. 3(a), with different frequency spacing. In the opposite case of $r_{Z}\to \infty$, a similar spectral structure is obtained but with the roles of the respective layers interchanged.

**FIG. 3.**
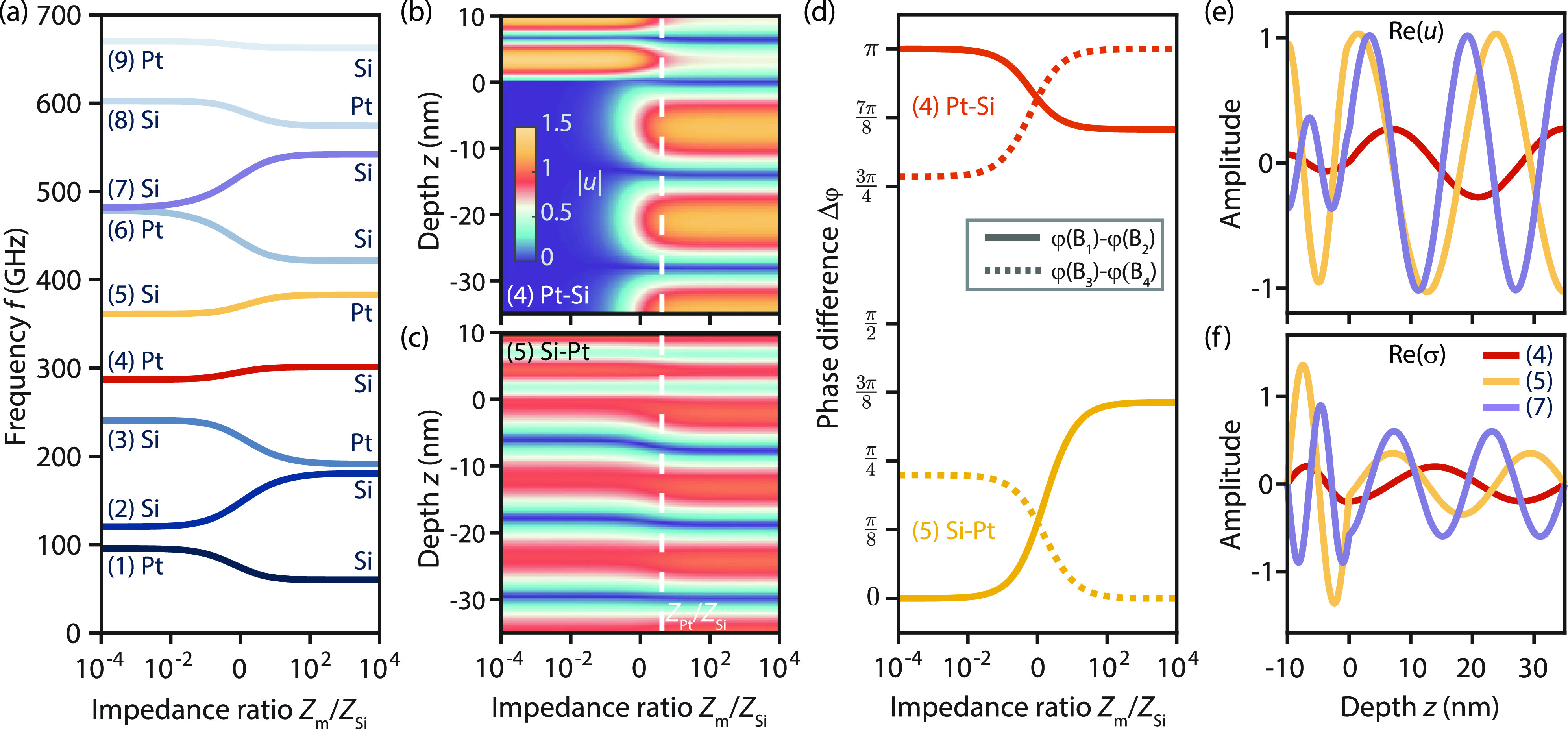
Acoustic mode profile and wave localization: (a) *n*th-order resonance frequencies depending on the impedance ratio $r_{Z}=Z_{m}/Z_{\text{Si}}$ of the two materials. (b) and (c) Absolute value of the displacement field, |u|, for branches (5) and (4), respectively. (d) Phase difference of the amplitude coefficients within each layer separately, depending on the impedance ratio *r_Z_* shown for branches (4) and (5) in red and yellow, respectively. (e) and (f) Instantaneous displacement *u* (e) and stress *σ* (f) for Pt/Si system with $r_{Z}=4.2$ as indicated by the white dashed line in (b) and (c). (d)–(f) Line colors are chosen consistently with (a).

Solving for the wave amplitudes at a given resonance frequency $f^{\left(n\right)}(r_{Z})$ (see the supplementary material S2) yields the corresponding displacement field, with the absolute wave amplitude shown in Figs. 3(b) and 3(c) for the resonance branches (4)-Pt–Si and (5)-Si–Pt. Both branches exhibit remarkably different spatial distributions of the wave amplitude. For the case shown in (c), atomic displacements of comparable amplitudes are observed in both layers for all impedance ratios. In contrast, for (4)-Pt–Si [Fig. 3(b)], the acoustic wave is localized in the upper material for small impedance ratios, shifting to the silicon layer with increasing impedance ratios.

The differences in the exemplary wave structures are caused by the acoustic boundary conditions [see Eq. (6) in the supplementary material] at the interface (*z* = 0), which result in a relation between the impedance ratio and the strain in both materials: $r_{Z}=\frac{Z_{m}}{Z_{\text{Si}}}=\frac{v_{\text{Si}}}{v_{m}}\frac{\varepsilon_{zz}^{\text{Si}}}{\varepsilon_{zz}^{m}}$. Specifically, the strain $\varepsilon_{zz}^{\text{Si}}$ ( $\varepsilon_{zz}^{m}$) at the interface vanishes for $Z_{m}\to 0$ ( $Z_{m}\to \infty$) at constant acoustic sound velocity ratios. For resonance frequencies governed by the material layer with a lower acoustic impedance, a sinusoidal standing wave with zero displacement at the layer interface and a displacement maximum at the open boundary is formed (as expected from the expression for the wave round trip times). For $r_{Z}\to 0$ and $r_{Z}\to \infty$, this effect leads to wave localizations in the metal layer and the silicon layer, respectively, as shown for branch (4) in Fig. 3(b). Similarly, wave localization is observed for all other (*n*)-Pt–Si branches (e.g., *n* = 1; see Fig. S3b in supplementary material). In contrast, for (*n*)-Si–Pt branches [e.g., for *n* = 5, shown in Fig. 3(c)], cosine-like standing waves are formed in silicon (metal) for $r_{Z}\to 0$ ( $r_{Z}\to \infty$). As a consequence of the (partial) wave transmission into the other layer, no distinct localization of the acoustic wave is observed.

The sinus- and cosine-like standing waves in the bilayer (in the limits of small/large *r_Z_*) become also evident from the phase difference of the forward and backward propagating wave components. As shown in Fig. 3(d), the sinusoidal behavior of (4) Pt–Si is apparent in the phase difference Δφ=π between the wave components in one of the layers. In contrast, Δφ=0 is observed for (5)-Si–Pt, as expected for a cosine-like standing wave field. Finally, equal phase angles occur in both layers in the impedance-matched case ( $\text{log}\;(r_{Z})=0$). For the specific case of the experimental Pt–Si-bilayer system (impedance mismatch $r_{Z}=4.2$), the instantaneous acoustic displacement and stress field at a chosen time *t* for selected resonance frequencies are shown in Figs. 3(e) and 3(f), respectively.

With the analytical acoustic mode description in mind, we now come back to the discussion of individual features apparent in the frequency maps shown in Figs. 2(a) and 2(b). First, the regions of vanishing mean strain [inclined/horizontal line-like features in Figs. 2(a) and 2(b), respectively] occur if integer multiples of the acoustic wavelength match the layer thickness. As shown in Fig. 2(b), such decreased mean strain amplitudes are found in the silicon layer for frequencies $f_{\text{Si}}=n\cdot \frac{v_{\text{Si}}}{H_{\text{Si}}}=n\cdot \frac{8433\;ms^{-1}}{35\;\text{nm}}=n\cdot 240.9\;\text{GHz},\;n\in N$, being independent of the metal sound velocity and therefore visible as violet shaded horizontal lines. For the metal layer [see Fig. 2(a)], the mean strain vanishes along diagonals with slopes proportional to the varied metal sound velocity.

Finally, the overall structure of the frequency maps can be understood by considering the resonances of the individual decoupled layers, corresponding to the horizontal and inclined lines in Figs. 2(a) and 2(b). Coupling of the resonances results in the appearance of avoided crossings, associated with a change in the resonance slope and in the character of the resonant mode structure. Such a behavior is strongly reminiscent of the coupling of diabatic quantum states and additionally has a close analogy to the resonance structure of an optical bilayer Fabry–Pérot interferometer (see the supplementary material S4).

For macroscopic acoustic resonators, a resonant mode picture is well established. In the case of localized nanoscale acoustic fields, a series of questions arises, for example, regarding the validity of continuum-theory boundary conditions at small length scales, the importance of the material interface for acoustic and electronic coupling or the impact of nanocrystallinity. The mode analysis presented here may serve as a reference model to gauge potential deviations arising from the nanoscale dimensions involved.

In conclusion, we determined the acoustic response of a nanoscale metal/semiconductor bilayer system upon femtosecond optical excitation, calculating the strain dynamics from linear-chain simulations and describing the acoustic field in terms of superimposed counter-propagating harmonic waves perpendicular to the sample surface. In the confined heterostructure and due to elastic boundary conditions, quantized phonon eigenmodes arise with frequencies in the gigahertz range, exhibiting strong couplings across the bilayer interface.

## SUPPLEMENTARY MATERIAL

See the supplementary material for further details on numerical and analytical models discussed in the main text.

## Data Availability

The data that support the findings of this study are available from the corresponding author upon reasonable request.
